# Meta-analysis, complexity, and heterogeneity: a qualitative interview study of researchers’ methodological values and practices

**DOI:** 10.1186/s13643-016-0366-6

**Published:** 2016-11-16

**Authors:** Theo Lorenc, Lambert Felix, Mark Petticrew, G J Melendez-Torres, James Thomas, Sian Thomas, Alison O’Mara-Eves, Michelle Richardson

**Affiliations:** 1 Centre for Reviews and Dissemination, University of York, York, YO10 5DD UK; 2 Department of Social and Environmental Health Research, London School of Hygiene & Tropical Medicine, 15-17 Tavistock Place, London, WC1H 9SH UK; 3 Warwick Evidence, Division of Health Sciences, Coventry, CV4 7AL UK; 4 EPPI-Centre, Social Science Research Unit, UCL Institute of Education, 20 Bedford Way, London, WC1H 0AL UK

**Keywords:** Complexity, Heterogeneity, Meta-analysis, Qualitative research, Systematic review methodology

## Abstract

**Background:**

Complex or heterogeneous data pose challenges for systematic review and meta-analysis. In recent years, a number of new methods have been developed to meet these challenges. This qualitative interview study aimed to understand researchers’ understanding of complexity and heterogeneity and the factors which may influence the choices researchers make in synthesising complex data.

**Methods:**

We conducted interviews with a purposive sample of researchers (*N* = 19) working in systematic review or meta-analysis across a range of disciplines. We analysed data thematically using a framework approach.

**Results:**

Participants reported using a broader range of methods and data types in complex reviews than in traditional reviews. A range of techniques are used to explore heterogeneity, but there is some debate about their validity, particularly when applied post hoc.

**Conclusions:**

Technical considerations of how to synthesise complex evidence cannot be isolated from questions of the goals and contexts of research. However, decisions about how to analyse data appear to be made in a largely informal way, drawing on tacit expertise, and their relation to these broader questions remains unclear.

## Background

In recent years, the challenge posed by complexity for systematic reviews and meta-analyses has been extensively discussed. Bringing together evidence on interventions which contain multiple components, or which might have been implemented differently in different studies, is inherently problematic. Beyond this, factors such as nonlinear dynamic pathways between intervention and outcome, feedback loops, emergent properties, and two-way interactions between the intervention and its contextual factors add to the complexity [1–3]. This poses a challenge for systematic reviewers of studies of complex interventions in relation to (1) framing the research question, (2) defining the intervention within the review, (2) searching for and locating relevant evidence, (3) standardising the selection of studies for a review, (4) synthesising data, and (5) generating robust overall conclusions of relevance to decision-makers [4].

An adequate engagement with complexity needs to move beyond the description of intervention components to include aspects of the population and setting and arguably also broader contextual factors [5, 6]. Standard systematic review frameworks such as PICOS (participants, interventions, comparators, outcomes, and study design) often do not address characteristics of setting, mechanisms of action or causal pathways that mediate outcomes, contextual factors that could have an impact on outcomes, and how the elements that contribute to complexity interact with each other [7, 8]. Moreover, systematic review authors are increasingly including a wider range of study designs that permit asking questions beyond “what works” to include questions of how it works, for whom, and in what circumstances [9]. Incorporating an understanding of complexity in review questions and methods may help to give a more complete understanding of the processes and outcomes of interventions [10]. Systematic reviews of complex data thus need to be “configurative” as much as “aggregative” [11], in the sense of exploring pathways and patterns of effect.

Many of the practical challenges of dealing with complexity come down to problems of heterogeneity—both statistical heterogeneity and substantive heterogeneity in terms of the aims, methods, and content of the studies populating a review. Traditionally, it was assumed that heterogeneity should be minimised to ensure the reliability of review findings. In the presence of complexity this may not be appropriate, since an adequate engagement with complex interventions and contexts demands the integration of heterogeneous types of data. In this context statistical heterogeneity is arguably to be expected, and may not be a useful indicator of problems with the data, but present opportunities for explanatory analysis. The challenge then is how to limit the boundaries of the review such that the engagement with heterogeneity can produce useful findings.

Negotiating between these hazards poses challenges for reviewers. In recent years, a range of methods have been developed which aim to engage constructively with heterogeneity, rather than seeing it purely as a problem to be minimised [7]. Quantitative methods such as network meta-analysis provide ways to synthesise heterogeneous data. New methodological approaches, such as realist synthesis, mixed- methods approaches which incorporate qualitative and other kinds of data along with quantitative synthesis, and Qualitative Comparative Analysis, are also promising ways of negotiating heterogeneity [12–14]. These newer approaches involve a shift of perspective, whereby heterogeneity is seen as a potential source of insights—about, for example, how the effectiveness of interventions varies according to context—rather than as noise obscuring the true message of the data.

However, although each of these approaches has an extensive literature of its own, there is limited general guidance on when to deploy these methods and the comparative strengths and limitations of each. This uncertainty may extend to whether a systematic review and meta-analysis should be conducted in a given context at all. The earlier debates between “lumpers” and “splitters” [15] have evolved into a complex methodological landscape in which many different methodologies may be applied to a particular body of data, each with its own challenges and limitations. The questions which then arise about the applicability of a method to a particular piece of research are often particularly intractable because they combine technical queries (about, for example, the data requirements of specific methods) with broader questions about the goals and contexts of the project in question (e.g. whether one should prioritise seeking a general measure of effect across a large area of practice or identifying differences and mediators). There is also an implicit tension between purely quantitative methods such as network meta-analysis and approaches such as realist synthesis which emphasise a theory-building approach to the integration of heterogeneous types of data (although theory may have a role to play in the use of quantitative techniques [16]). Views on all these questions may vary according to the context of the research, for example whether the project aims to answer practical or policy questions or is conceived as “pure” scientific inquiry. Researchers’ methodological practices—by which we mean both the macro-level choice of “a” method appropriate to a given question and the micro-level choices involved in applying the method—may thus be informed by the values which inform broader research agendas.

All these methods and approaches have valuable contributions to make to the synthesis of complex and heterogeneous data. However, the increasing range of methodological choices open to researchers poses its own challenges. The aim of this paper is to investigate researchers’ understanding of their own practices in evidence synthesis, and the social, cultural and individual factors which may structure these practices. To this end, we explore the views of researchers working in systematic review and meta-analysis on complexity and heterogeneity and their experiences working with complex data. We purposively sampled participants for the diversity of their experience and disciplinary affiliations, with sampling informed by thematic saturation. With a few exceptions [17, 18], the attitudes and practices of researchers in this field have not been widely researched. We aimed to explore the issues around the practice of systematic review and meta-analysis of complex data, so as to complement formal methodological guidance, by collecting qualitative data on researchers’ views and experiences.

## Methods

Participants (*N* = 19) were researchers with substantial experience in systematic review and/or meta-analysis. Most participants were based in the UK. We aimed specifically to recruit participants who had undertaken reviews and meta-analyses on complex topics or including heterogeneous data. We sampled purposively for diversity in disciplinary affiliation and theoretical approach. In particular, we aimed to recruit participants working in fields where systematic review and meta-analysis are relatively new, or not widely used, rather than focusing exclusively on disciplines such as healthcare or criminology where these approaches are well-established. Table 1 gives more details about the participants.

**Table 1:** Participants’ characteristics

Participant	Discipline	Country
1	Environment	UK
2	Software engineering	UK
3	Software engineering	UK
4	Statistics	UK
5	Psychology	Canada
6	Public health	UK
7	Economics	UK
8	Public health	UK
9	Economics	UK
10	Public health	UK
11	Psychology	UK
12	Public health	UK
13	Social care	UK
14	Psychology	UK
15	Sociology	UK
16	Environment	UK
17	Public policy	UK
18	Public health	UK
19	Economics	USA

Semi-structured individual interviews were conducted face-to-face or by telephone, using an interview guide which covered topics including guidance used to inform systematic review methodology, framing research questions, data synthesis, heterogeneity, complexity, and publication bias. All interviews were tape-recorded and transcribed. Data analysis used a framework approach, which is a more structured method than purely inductive forms of analysis based on grounded theory [19]. The initial coding frame was based on the interview schedule, which was used to produce high-level codes reflecting the domains of interest to the study. A second phase of coding involved the development of subcodes within this framework, which were developed inductively from the data. In a final phase, the transcripts were re-read for any emergent themes not captured by the framework. Coding was carried out by two researchers working in tandem and then discussed with the other members of the research team. Ethical approval was obtained from the London School of Hygiene & Tropical Medicine (LSHTM) Ethics Committee (Ref: 8545).

## Results

Nineteen participants participated in the interviews. Three interviews were conducted face-to-face while the remaining interviews were conducted by telephone. All the participants were affiliated to an academic institution, and all but two were based in the UK. The first three interviews were conducted by two authors (two by LF and MP, one by LF and ST) in order to pilot the interview guide; one further later interview was conducted by two authors (LF and JT) to ensure adequate methodological expertise in the interviewee’s field. All other interviews were conducted by one author (LF) alone. The duration of the interview ranged from 27 to 59 min.

### Challenges in conducting systematic reviews of complex evidence

Participants defined “complexity” in a number of ways. One offered a summary of the levels at which complexity may arise: “One, it could be the level of the intervention. Two it could be the level of the context and three it could be at the level of the types of data that you need to answer your question” (participant 11). Interventions themselves may be complex due to the inclusion of components at multiple levels: “something that involves different numbers of people or different types of people all working at different levels” (17). Contextual complexity may arise because of how implementation differs between contexts or how contextual factors mediate intervention effect: “what modifies the relationship between intervention and its outcomes, that might be a whole load of things to do with the intervention, but it might be […] to do with a whole load of things that have got nothing to do with the intervention” (10). Finally, complexity may arise from the need to synthesise multiple data types and in some cases from the need to integrate a wider range of data than would be considered in a traditional review.

Two participants argued that there is no clear line dividing complex from non-complex interventions and suggested that complexity is more the rule than the exception: “if you work with social interventions you can be almost certain it’s complex. It’s just a matter of degree to how complex it is” (17). As one participant observed, the determining factor is perhaps less the presence of complexity at any one of these levels than the inevitability of unpredictable interaction between them.

Participants mentioned a number of challenges in conducting systematic reviews of complex evidence, including limitations in research databases, insufficient good-quality primary evidence, a lack of resources or skills to conduct reviews, difficulty in accessing funding, and pressures from research users, for example to maximise breadth of inclusion criteria. Some of these issues were felt to be particularly acute in fields where systematic review is a relatively recent introduction, such as software engineering. Participants identified a number of underlying characteristics of complex questions or bodies of evidence which give rise to these challenges, including the importance of context (in particular the impossibility of filtering out or controlling for exogenous factors which may influence the effectiveness of an intervention), variations in the fidelity of implementation of interventions, and multiple intervention components (or multiple interventions within a single review). One participant described how the evidence based on complex interventions reflects the history of policy implementation, increasing the difficulty of interpreting the evidence: “all social interventions have a history where they tend to have been tried and tried again and according to how history’s treated them, interventions come out differently” (15). This participant also observed that the policy context may frequently be an irreducible part of the effectiveness of interventions—for example, outcomes evaluated by studies may also be used as performance indicators by service managers.

### Guidance used to inform systematic review methodology

Several participants discussed the use of guidance in conducting reviews. For example, participants conducting a realist synthesis referred to the RAMESES guidance (Realist And Meta-narrative Evidence Syntheses: Evolving Standards). The Cochrane handbook and the Campbell guidance emerged as the most popular reference used by several participants to inform their systematic review methodology, for both Cochrane and non-Cochrane reviews. Participants from disciplines such as environmental science and software engineering mentioned that these fields have developed their own guidance drawing inputs from the Cochrane and Campbell collaboration. Although Cochrane’s Methodological Expectations of Cochrane Intervention Reviews (MECIR) conduct standards and Preferred Reporting Items for Systematic Reviews and Meta-Analyses (PRISMA) guidelines are primarily produced to guide the reporting of systematic reviews, participants also reported using them as a quality check of methods. Table 2 lists the resources mentioned by the participants to inform their systematic review methodology.

**Table 2:** Main guidance used by participants

Cochrane Handbook [30]
Campbell Collaboration guidance [31]
RAMESES guidance on realist synthesis [32]
Cochrane MECIR conduct standards [33]
PRISMA Statement [34]
Cooper et al., *Handbook of Research Synthesis* [35]
Petticrew and Roberts, *Systematic Reviews in the Social Sciences* [36]
Littell et al., *Systematic Reviews and Meta-analysis* [37]
Saini and Shlonsky, *Systematic Synthesis of Qualitative Research* [38]
ESRC guidance on narrative synthesis [39]
CRD guidance [40]
Cochrane EPOC guidance [41]
SCIE guidance [42]

Some participants felt that available guidance did not adequately address all their questions: “a lot of the methods we just have to work out” (17). One other participant suggested that once the basic principles of reviews are internalised, it may be possible to dispense with specific recommendations such as the PICO framework or exhaustive searching: “I’m not particularly wedded to the exhaustiveness once you move outside of trials or predefined protocols or predefined search strategies as long as you can see what [you’ve] done” (16).

### Review questions and frameworks

The formulation of the review question was frequently identified as important in conducting reviews on complex questions. Maintaining a degree of breadth in the review question was seen as important: three participants suggested that overly specific questions may be inappropriate because of the risk of producing a review which is so narrow as to be irrelevant to practice or empty: “you’ve restricted the question to a point where actually nobody’s interested in the answer any longer, it’s so tiny and so narrow and so restricted” (13). Seven participants argued that reviews which engage with complexity need to go beyond questions of the effectiveness of interventions to look at how and why interventions work, for whom, and in what contexts: “social interventions and complex interventions are embedded within systems and are influenced by other factors and these are dynamic in themselves and how they deliver outcomes and interact with individuals or communities. So it doesn’t make sense to just look at whether or not there is an effect” (8).

As some of these participants made clear, this implies a more inclusive approach than is often practiced with respect to outcomes and study designs. The inclusion of qualitative studies in mixed methods reviews was mentioned by three participants as a potentially promising way to illuminate these broader contextual factors. However, some caution was expressed here: one participant suggested that reviewers without specialist training may be ill-equipped to make use of qualitative evidence and two others that there is a lack of clear guidance on synthesising qualitative research and of formal tools for managing heterogeneity.

There was some disagreement among participants as to how and whether review questions should explicitly include the exploration of heterogeneity; this disagreement is not purely methodological but relates to broader questions of how reviews should inform policy and practice. Two participants argued that the exploration of heterogeneity should be secondary to identifying what is common to the diverse research findings. “[O]f course, we would like to understand the heterogeneity of the treatment effect […] But in the vast majority of these policy settings, at least in the area that I’m working, people just understanding the average effect and letting that help inform their policy decisions would be an improvement, in terms of the use of evidence” (7). By contrast, one participant argued that heterogeneity should be conceptualised as the primary focus of synthesis, in the sense that “why an intervention varies in its effectiveness” is often of more interest than “classical” questions of whether or not it is effective: “although the questions might be classically framed, quite often the interest is in understanding the heterogeneity that we find across studies” (1).

A particular problem here, mentioned by three participants, is that, in practice, heterogeneity is often dealt with post hoc and not adequately theorised. This is not only statistically questionable but prevents real insight into the data: “if you don’t have any theory about how you’re going to explore the heterogeneity […] then […] exploring it just means you spent more time doing it and not learning much more” (17). Two participants suggested that the question of whether the synthesis of heterogeneous data is appropriate and meaningful cannot be adequately answered by researchers alone but requires engagement with broader communities of practitioners and research users: “I would always say go to the people on the ground and say, is it appropriate?” (11). These data suggest that in the presence of complexity, the management of heterogeneity needs to both be considered at the level of the review question and on an ongoing basis throughout the review and cannot be reduced to a technical issue of data analysis methodology.

Logic models—“diagrams on one page that try to articulate in a visual way some of the complex hypothetical pathways for impact” (8)—were mentioned by four participants as a potentially useful tool in the exploration of complex data. Participants reported that logic models can be useful both in the early stages of a review, to refine review questions and methods, and in the later stages of data synthesis to visualise relationships between large numbers of studies and variables. They found logic models particularly useful for identifying moderators of intervention effect to be explored by the synthesis and as an aid to integrating qualitative and process evidence with data on effectiveness.

The use of programme theories was also mentioned by two participants. One in particular drew on realist evaluation theory to argue that the programme theory should be regarded as the unit of analysis rather than the intervention. “Interventions aren’t the basic unit of analysis. The programme theory is. […] Policies and interventions and programmes began, begin in the thought process where somebody says, well here’s the problem, this is what I think is the nature of the problem, this is what I think is the solution, this is how people, this is how I think people are going to respond to the resources that we provide” (15).

### Meta-analysis

Several factors were noted by participants as affecting the decision as to whether meta-analysis is appropriate, including the statistical validity of data, similarity of intervention components and participants across studies, and the interpretability of outcome measures. One participant suggested that the number of studies available for synthesis may also be a concern and estimated that at least ten studies are generally required to produce certainty in the results. This last point aside, participants generally did not specify clearly defined thresholds or processes for making the decision: rather, it is a matter for judgement informed by a range of factors which may vary in importance depending on the case.

There was some disagreement about how to explore heterogeneity in meta-analyses: two participants recommended conducting meta-analysis at the outset, if the data permits, and then exploring the heterogeneity from the data by pursuing additional analysis such as meta-regression, while one argued that this should only be done if there is an a priori plan about what constitutes heterogeneity and how it will be explored. One participant reported using a staged process: “you stratify the analysis first, and then if you don’t see any significant differences in effects then you can pool them because that obviously gives you a lot more power to do further sub-group analysis” (9).

In terms of methods for meta-analyses, several participants used standard pairwise meta-analysis. This was seen to have several advantages, for example the ease of interpretation of forest plots: “it’s much easier to present things graphically and just discuss a weighted average” (4). Three participants also discussed the use of network meta-analysis as a potentially useful tool in the synthesis of complex data, due to the ability to conduct syntheses in the absence of data to conduct a direct comparison. However, some participants felt that there are still limitations on its use. One observed that network meta-analysis relies on a certain level of homogeneity at the level of population and intervention content, and another suggested that “it’s just compounding the uncertainty we had with the original comparisons” (11).

Meta-regression was also discussed by three participants, with one in particular arguing strongly that, given a sufficient quantity of data, meta-regression alone can largely solve any problem presented by heterogeneity and that the challenge of heterogeneity is wholly reducible to the question of whether it can be statistically accounted for: “heterogeneity *per se* doesn’t bother me, unaccounted for heterogeneity does” (19). That is, meta-regression is thought to allow reviewers to incorporate an understanding of the impacts of heterogeneity and gain a more complete picture of the evidence. There is considerable judgement involved in choosing moderator variables to include in a meta-regression, drawing on both broad knowledge of the field in question and previous experience with the method.

Some data suggest that the question of whether to utilise tools such as meta-regression may differ depending on disciplinary or institutional affiliations. Two participants alluded to a debate between Campbell-based researchers who make extensive use of methods such as meta-regression to manage heterogeneity and Cochrane-based researchers who often choose not to meta-analyse in the presence of heterogeneity. However, they took opposed positions on this debate: one sympathised more with the latter position and expressed concern about researchers being “steamrollered” (11) into inappropriate analyses, while the other argued that researchers have an obligation to anticipate and manage heterogeneity within the review process, rather than simply refusing to undertake syntheses of heterogeneous data.

### Publication bias

Participants were also asked specifically for their views on publication bias. Two observed that there are problems with standard statistical tests such as the funnel plot, which may show asymmetry for reasons unrelated to publication bias, and that these problems have not been convincingly addressed. One participant suggested that researchers’ uncertainty on how to manage publication bias is well-founded: “I’m quite sympathetic to the problems of researchers who, the synthesisers who come up […] against publication bias, but really don’t know how to handle it, because I think it is difficult to handle adequately” (4). While publication bias can be lessened by searching for grey literature, it cannot be entirely removed, since in some cases, study findings may not even reach the grey literature but remain “in the file drawer.” Generally, these issues seem not to be specific to complex or heterogeneous data, however, but apply to reviews across the board.

## Discussion

Our findings indicate that systematic reviewers face a range of challenges in dealing with complex and heterogeneous data. Those working in fields such as environmental science or engineering, where systematic reviews and meta-analyses are relatively recent introductions, may face extra difficulties, for example a lack of reliable primary studies. However, many of the key issues raised appear to be consistent across disciplines. Systematic reviewers use a range of guidance and tools but take a pragmatic attitude to them rather than applying them mechanically. A common theme is that reviews on complex questions need to take an inclusive approach, integrating contextual data and aiming to explore heterogeneity rather than explaining it away. A range of approaches, both quantitative and mixed- methods, are used to extend the scope of “traditional” meta-analysis; all these methods have potential challenges and demand a degree of judgement and experience in their application. Researchers face the task of negotiating a pathway between the extremes of either applying methods in a rigid and uninformative way, or taking an excessively lax approach which compromises the integrity of the methods and gives misleading results.

With one or two exceptions, participants in this study did not report reliance on a single overarching methodology to inform the conduct of reviews and meta-analyses. Participants found that traditional methods for making a heterogeneous evidence base more tractable, such as hierarchies of evidence, are often not appropriate when dealing with complex questions. A more inclusive approach to the evidence will often be more productive: techniques such as logic models are valued because they facilitate such an approach, as much as for their inherent merits. Several participants reported a pragmatic attitude to existing guidance and methods and a willingness (or necessity) to find novel answers to unexpected methodological challenges. These findings suggest that researchers decide which aspects of methodology are relevant to a particular question or data set using their own expertise and judgement, in a form of *bricolage* [20] which may not be avowed in research reports. More broadly, they point to the importance of tacit or “craft” knowledge [21] among researchers in determining the applicability of methodological precepts to a given case. As sociologists of knowledge have long argued, the labour of constructing and applying categories or structures always involves negotiating with ambiguity and cannot be separated from social and ethical values [22, 23]. Research synthesis—the “engagement between resisting readers and resistant texts,” as Sandelowski evocatively describes it [24]—is no exception to this general point, particularly in the presence of complexity and heterogeneity. Reflexive accounts of the process of meta-ethnography have drawn attention to the tension between the production of synthetic constructs and the recognition of multiplicity [25, 26]; our findings suggest that a similar tension may underlie decisions about syntheses of quantitative evidence.

These decisions include, for example, whether meta-analysis is appropriate at all in a given case, and what methods should be used, particularly whether heterogeneity should be explored post hoc. Participants recognised that these decisions depend not only on the nature of the data and the broader context of the analysis (e.g. writing for publication as against preliminary exploratory work) but also on individual researchers’ skills and perspectives. The informality of decisions on meta-analysis suggests that social and cultural factors may play some role in structuring them. Our findings provide some examples of how these are informed by the values held within distinct “epistemic cultures” [27], for example the split between Cochrane- and Campbell-affiliated researchers regarding the use of meta-regression. Disciplinary differences may also play a role, although the generally interdisciplinary nature of research teams and individual careers in most of the fields studied mean it is hard to generalise about this.

At a more abstract level, participants’ responses can be seen to reflect two ontological perspectives, whereby a research project can be seen either as a series of analyses conducted on a body of data or as an intervention in a social and political context (either critically or constructively, for example as a decision-making tool). There is a sense in our data that technical questions about the analysis of quantitative data are largely isolated from questions about the intended utilisation of research findings, the role of stakeholders, or the political and historical contexts of research projects.

For example, we noticed two debates that ostensibly should have informed each other. First, the debate about whether it is more useful to focus on yes/no questions of whether an intervention is effective, or to explore how and for whom it is effective, raises broad questions about the place of research evidence in policymaking and about whose perspectives should inform the conduct of research. Second, the debate about how best to incorporate discussion of heterogeneity into meta-analyses, and whether a priori hypotheses are necessary for this, raises questions about the methodological aspects of statistical synthesis. Yet, despite their clear mutual relevance, these debates appear to have proceeded on parallel tracks, with little reflection either on how social and epistemological questions might impact the application of statistical methods or about how developments in methods might change the relation of research to policymaking or to broader public concerns.

One possible practical implication is that it may not be possible to have a single benchmark against which to assess quality of systematic reviews, and that the absence of formalised guidance on key review decisions reflects this impossibility. This is because different perspectives as to the methodological pathways and purposes embedded in systematic reviews, and the values embedded in these perspectives, will yield different criteria for “quality.” For example, the view of systematic reviews that privileges review-as-analysis suggests that reviews are a tool for enlightenment, irrespective of their practical implications. Thus, high-quality reviews in this view will foreground robustness of statistical methods and transparency of method over relevance and theoretical engagement. This is commensurate with the view that systematic reviews themselves cannot make recommendations for action. On the other hand, the view of systematic reviews that privileges review-as-intervention implies an instrumental view of research (which one might see in the context of Nowotny et al.’s “mode 2 knowledge” [28] or Funtowicz and Ravetz’ “post-normal science” [29]). High-quality “instrumental” reviews may share the same traditional markers of quality but will additionally provide information that is ready-to-wear and that advances understanding about the intervention itself rather than just its effectiveness.

This is not to advocate a nihilistic view of quality in evidence synthesis. Rather, it is to advocate a reflexive understanding on the part of reviewers of what the goal of undertaking a systematic review is. Such reflexive understanding would also help to promote the transparency of the review process as a whole. Of course, researchers do currently take into account the social or practical implications of research in making decisions about meta-analysis, and it is likely that views about the contexts and purpose of research projects do influence these decisions, as well as expert judgement based on technical considerations. However, the former appear to be rarely discussed or argued for explicitly. This raises the possibility that such broader concerns may often influence the conduct of reviews in ways which are not fully transparent.

This study was intended as an exploratory investigation and is certainly not conclusive. The sample was fairly small and focused on researchers working in the UK: although we aimed to recruit participants with a diverse range of experience, participants may not be reflective of all work in reviews and meta-analysis, and some disciplines are under-represented. While we aimed to integrate ideas from different disciplines, the theoretical framework of the project as a whole (particularly the key idea of complexity) draws primarily on public health and health service research. Other research traditions (e.g. in education or psychology) have their own histories of engaging with complexity and heterogeneity which may not be reflected in our data. Further work with a broader range of participants, representing different research traditions, would be valuable. In addition, data derive only from interviews, and we kept the questions at a general level so as to elicit broad views about methods. It would be illuminating to pursue these questions with reference to more specific projects and decisions, perhaps using participant observation or “think-aloud” methods which would enable exploration of how researchers actually work.

## Conclusions

This study finds that researchers use a wide range of methodological approaches to the synthesis of complex and heterogeneous data, including statistical approaches such as network meta-analysis and meta-regression, and non-statistical approaches such as logic models, realist synthesis and mixed methods synthesis. There is considerable debate about the merits of all of these and the circumstances in which they are applicable. In practice, researchers take a pragmatic approach, using their judgement and experience to choose which methods are appropriate in a particular case. These choices may be influenced by many different factors to do with both the studies populating the review and the broader contexts and purpose of the research. However, with some exceptions, technical decisions about quantitative analysis appear to be pursued in isolation from questions about the substantive meaning of the data and the social, political, and practical contexts of the synthesis. A broader dialogue among researchers, bringing together these two kinds of question, would help to promote transparency of decision-making throughout the research process.

## Abbreviations

CRD: Centre for Reviews and Dissemination
EPOC: Effective Practice and Organisation of Care
ESRC: Economic and Social Research Council
LSHTM: London School of Hygiene & Tropical Medicine
MECIR: Methodological Expectations of Cochrane Intervention Reviews
PICO: Population, intervention, comparator, outcomes
PRISMA: Preferred Reporting Items for Systematic Reviews and Meta-Analyses
RAMESES: Realist And Meta-narrative Evidence Syntheses: Evolving Standards
SCIE: Social Care Institute for Excellence
